# Beyond bioavailability: dual-source iron improves absorption and cellular handling

**DOI:** 10.3389/fnut.2026.1860836

**Published:** 2026-07-01

**Authors:** Sara De Martin

**Affiliations:** Department of Pharmaceutical and Pharmacological Sciences, University of Padova, Padova, Italy

**Keywords:** heme iron, iron bioavailability, non-heme iron, nutraceutical supplements, tolerability

## Abstract

**Figure:**
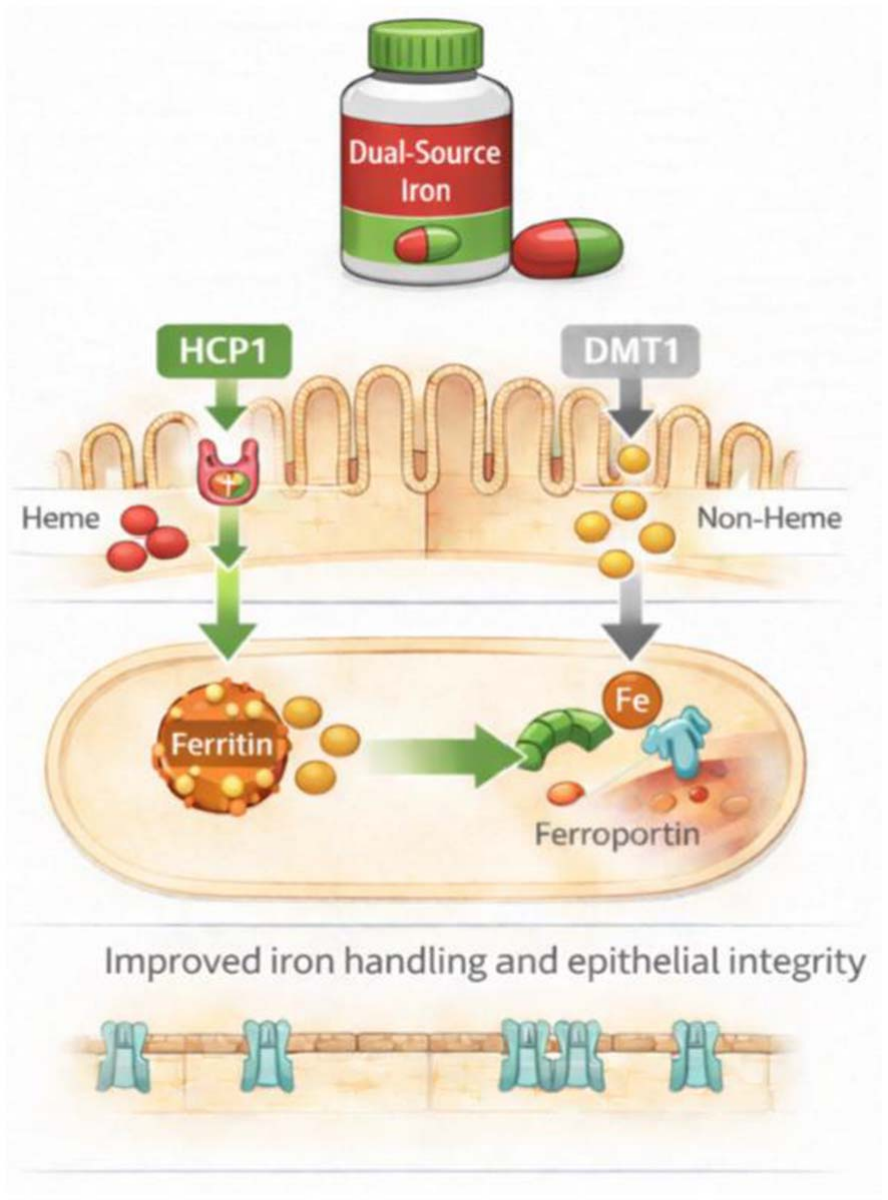
Dual-source iron enhances intestinal absorption and cellular handling through complementary pathways. Schematic representation of the mechanisms underlying the improved performance of dual-source iron formulations containing both heme and non-heme iron. In the intestinal epithelium, heme iron is absorbed via heme carrier protein 1 (HCP1), while non-heme iron is taken up through divalent metal transporter 1 (DMT1), enabling the engagement of complementary uptake pathways. Following intestinal uptake, iron is efficiently processed within enterocytes, as indicated by increased ferritin-mediated intracellular storage and ferroportin-dependent export into the circulation. This coordinated handling supports improved systemic iron availability. Importantly, the combined engagement of these pathways is associated with preserved epithelial integrity, likely due to reduced levels of unabsorbed luminal iron. Overall, dual-source iron formulations integrate enhanced uptake and efficient intracellular processing with maintenance of intestinal barrier function, providing a mechanistic basis for improved iron handling.

Dual-source iron enhances intestinal absorption and cellular handling through complementary pathways. Schematic representation of the mechanisms underlying the improved performance of dual-source iron formulations containing both heme and non-heme iron. In the intestinal epithelium, heme iron is absorbed via heme carrier protein 1 (HCP1), while non-heme iron is taken up through divalent metal transporter 1 (DMT1), enabling the engagement of complementary uptake pathways. Following intestinal uptake, iron is efficiently processed within enterocytes, as indicated by increased ferritin-mediated intracellular storage and ferroportin-dependent export into the circulation. This coordinated handling supports improved systemic iron availability. Importantly, the combined engagement of these pathways is associated with preserved epithelial integrity, likely due to reduced levels of unabsorbed luminal iron. Overall, dual-source iron formulations integrate enhanced uptake and efficient intracellular processing with maintenance of intestinal barrier function, providing a mechanistic basis for improved iron handling.

## 1 Introduction

Iron deficiency remains one of the most prevalent nutritional disorders worldwide and continues to represent a major public health concern across diverse populations. Despite the widespread availability of oral iron supplements, their clinical efficacy is often limited by variable intestinal absorption and poor gastrointestinal tolerability (1, 2). These limitations reflect the tightly regulated nature of iron homeostasis, in which absorption is finely tuned according to physiological demand and influenced by the chemical form of iron. Non-heme iron, which constitutes the majority of dietary and supplemental iron, is limited by multiple luminal and intracellular constraints, including pH-dependent solubility, oxidation state, and competition with dietary inhibitors. In contrast, heme iron is absorbed through a distinct and more efficient pathway that is relatively independent of these factors (3). The limited efficacy of many conventional oral formulations highlights a broader challenge in iron supplementation, i.e., improving bioavailability without increasing the burden of unabsorbed luminal iron. Indeed, excessive intraluminal iron may contribute to oxidative stress, mucosal irritation, and alterations in gut microbial composition, mechanisms increasingly implicated in gastrointestinal intolerance and reduced treatment adherence. These considerations suggest that the optimization of iron therapy should not rely exclusively on increasing elemental iron exposure, but also on improving the physiological efficiency of absorption and intracellular handling. Within this framework, growing attention has been directed toward formulations that combine heme and non-heme iron sources in the attempt to engage complementary uptake pathways. However, the mechanistic basis through which dual-source formulations may influence the coordinated processes of iron uptake, intracellular storage, and export within complex biological systems remains insufficiently characterized. In this context, a recent study by Parini and collaborators (4) provides a useful mechanistic framework to explore how dual-source formulations may influence iron absorption dynamics and cellular processing.

## 2 Strengths and mechanistic insights

A key strength of this study lies in the use of a validated Caco-2 intestinal barrier model, an approach widely recognized and endorsed by regulatory agencies such as the FDA and EMA for assessing oral drug absorption and bioavailability (5, 6). This is particularly relevant in the context of iron supplementation, where absorption is inherently limited and subject to high inter-individual variability (7). Within this framework, the authors compared dual-source formulations combining heme and non-heme iron at different elemental doses with conventional non-heme supplements and inorganic salts. By leveraging a model commonly used in pharmaceutical development, the study moves beyond descriptive *in vitro* observations and generates findings that are more directly informative for intestinal physiology. The results indicate that dual-source formulations enhance iron uptake and intracellular handling while preserving epithelial integrity. Functional assessments and preservation of tight junction protein expression suggest that these formulations do not compromise epithelial barrier integrity. This aspect is clinically relevant because excessive luminal iron exposure has been associated with oxidative stress, mucosal irritation, and microbiota alterations, all of which may contribute to the gastrointestinal symptoms that frequently limit adherence to oral iron therapy (8). From a mechanistic perspective, the study provides evidence that combining heme and non-heme iron enables the engagement of complementary uptake pathways. The upregulation of both the primary non-heme transporter DMT1 (9) and the apical heme transporter HCP1 (10) suggests that dual-source formulations may exploit parallel absorption mechanisms. This dual engagement may represent a physiologically relevant strategy to enhance iron uptake beyond what can be achieved by single-source formulations, although the extent to which transporter upregulation predicts improved clinical efficacy remains uncertain. In addition, increased expression of ferritin (11) and ferroportin (12) indicates effective intracellular storage and export of iron. These findings support the concept that absorbed iron is not only taken up more efficiently but also appropriately processed and mobilized for systemic use. Notably, formulations containing both iron sources exhibited a more sustained absorption profile over time, suggesting that heme iron may contribute to improved temporal dynamics of iron availability.

## 3 Limitations and interpretations

While the mechanistic findings are compelling, their translational relevance requires careful contextualization before clinical extrapolation. Caco-2 models provide a robust platform for studying epithelial transport processes, yet they cannot reproduce the systemic feedback mechanisms governing iron homeostasis, particularly the hepcidin–ferroportin axis, which critically regulates intestinal iron absorption under inflammatory and iron-replete conditions. Moreover, increased transporter expression does not necessarily predict improved long-term clinical efficacy, particularly under conditions in which iron absorption could be physiologically suppressed. An additional consideration is that the formulations differed not only in iron source but also in elemental dose and composition, making it difficult to disentangle the specific contribution of dual-source delivery from formulation-dependent effects. These limitations do not diminish the relevance of the findings but rather frame them as mechanistic signals that require validation in clinically meaningful settings.

## 4 Discussion

The findings discussed here support a broader conceptual shift in how iron supplementation strategies are approached within nutritional science. Rather than focusing exclusively on increasing the quantity of administered iron, dual-source formulations emphasize the importance of engaging physiologically distinct absorption pathways to enhance overall efficiency. This perspective aligns with a more integrated view of nutrient handling, in which uptake, intracellular processing, and epithelial integrity are considered interdependent components of effective supplementation. By improving the efficiency of intestinal iron handling, dual-source formulations may also contribute to a more favorable gastrointestinal tolerability profile, potentially reducing some of the limitations associated with conventional oral supplementation. From a translational standpoint, this approach has the potential to address two persistent challenges in oral iron therapy, i.e., inconsistent absorption and poor tolerability. These advantages may be particularly relevant in populations with increased iron requirements or impaired absorption, including women of reproductive age, individuals with inflammation-associated iron deficiency, and physically active populations. However, the extent to which these mechanistic benefits translate into clinically meaningful outcomes remains to be established. This may be particularly relevant in conditions characterized by impaired iron utilization or inflammation-associated alterations in iron homeostasis, where systemic regulatory pathways such as hepcidin signaling may substantially influence treatment response. Future research should prioritize well-designed clinical trials that directly compare dual-source and conventional formulations under conditions that reflect real-world use. Future clinical studies should integrate not only conventional biomarkers of iron status, but also mechanistic readouts linked to tolerability and intestinal homeostasis, including hepcidin dynamics, markers of oxidative stress, inflammatory mediators, and indicators of gut barrier function or microbiota alterations. Such approaches may help identify responder profiles and clarify whether the improved epithelial handling observed *in vitro* translates into clinically meaningful benefits. In conclusion, dual-source iron formulations represent a promising evolution toward a more physiology-driven approach to iron supplementation. By integrating complementary absorption pathways with efficient intracellular handling and preservation of epithelial integrity, this strategy may contribute to the development of more effective and better-tolerated nutritional interventions aimed at improving patient outcomes.

## References

[1] Kolarš B Mijatović Jovin V Živanović N Minaković I Gvozdenović N Dickov Kokeza I . Iron deficiency and iron deficiency anemia: a comprehensive overview of established and emerging concepts. Pharmaceuticals. (2025) 18:1104. doi: 10.3390/ph18081104 40872496 PMC12389209

[2] Hamarsha Q Kawtharany H Choaib A Azzam M Khawandi J Alkasrawi H . Prevalence of iron deficiency with or without anemia in adults: a systematic review and meta-analysis. Blood. (2025) 146:6468. doi: 10.1182/blood-2025-6468

[3] Gallo Ruelas M Alvarado-Gamarra G Aramburu A Dolores-Maldonado G Cueva K Rojas-Limache G . A comparative analysis of heme vs non-heme iron administration: a systematic review and meta-analysis of randomized controlled trials. Eur J Nutr. (2025) 64:51. doi: 10.1007/s00394-024-03564-y 39708071 PMC11663168

[4] Parini F Galla R Mulè S Musu M Uberti F. improved iron uptake and metabolism through combined heme and non-heme iron supplementation: an *in vitro* study. Biomedicines. (2025) 14:43. doi: 10.3390/biomedicines14010043 41595579 PMC12838038

[5] FDA. M9 Biopharmaceutics Classification System-Based Biowaivers. Available online at: https://www.fda.gov/media/148472/download (Accessed June 9, 2026).

[6] EMA. ICH Guideline M9 on Biopharmaceutics Classification System Based Biowaivers. Available on line at: https://www.ema.europa.eu/en/documents/scientific-guideline/ich-m9-biopharmaceutics-classification-system-based-biowaivers-step-5_en.pdf (Accessed June 9, 2026).

[7] Piskin E Cianciosi D Gulec S Tomas M Capanoglu E. Iron absorption: factors, limitations, and improvement methods. ACS Omega. (2022) 7:20441–56. doi: 10.1021/acsomega.2c01833 35755397 PMC9219084

[8] Malesza IJ Bartkowiak-Wieczorek J Winkler-Galicki J Nowicka A Dzieciołowska D Błaszczyk M . The dark side of iron: the relationship between iron, inflammation and gut microbiota in selected diseases associated with iron deficiency anaemia—a narrative review. Nutrients. (2022) 14:3478. doi: 10.3390/nu14173478 36079734 PMC9458173

[9] Yanatori I Kishi F. DMT1 and iron transport. Free Radic Biol Med. (2019) 133:55–63. doi: 10.1016/j.freeradbiomed.2018.07.020 30055235

[10] Latunde-Dada GO Takeuchi K Simpson RJ McKie AT. Haem carrier protein 1 (HCP1): expression and functional studies in cultured cells. FEBS Lett. (2006) 580:6865–70. doi: 10.1016/j.febslet.2006.11.048 17156779

[11] Srivastava AK Reutovich AA Hunter NJ Arosio P Bou-Abdallah F. Ferritin microheterogeneity, subunit composition, functional, and physiological implications. Sci Rep. (2023) 13:19862. doi: 10.1038/s41598-023-46880-9 37963965 PMC10646083

[12] Ward DM Kaplan J. Ferroportin-mediated iron transport: Expression and regulation. Biochim Biophys Acta. (2012) 1823:1426–33. doi: 10.1016/j.bbamcr.2012.03.004 22440327 PMC3718258

